# Cervical spine and muscle adaptation after spaceflight and relationship to herniation risk: protocol from ‘Cervical in Space’ trial

**DOI:** 10.1186/s12891-022-05684-0

**Published:** 2022-08-13

**Authors:** Daniel L. Belavy, Gabriele Armbrecht, Kirsten Albracht, Helena Brisby, Deborah Falla, Richard Scheuring, Roope Sovelius, Hans-Joachim Wilke, Kajsa Rennerfelt, Eduardo Martinez-Valdes, Michail Arvanitidis, Fabian Goell, Bjoern Braunstein, Svenja Kaczorowski, Vera Karner, Nitin Kumar Arora

**Affiliations:** 1 Department of Applied Health Sciences, Division of Physiotherapy, Hochschule für Gesundheit (University of Applied Sciences), Gesundheitscampus 6-8, 44801 Bochum, Germany; 2 Center for Muscle and Bone Research, Charité – University Medicine Berlin, Hindenburgdamm 30, 12203 Berlin, Germany; 3 Department of Medical Engineering and Technomathematics, Aachen University of Applied Sciences, Aachen, Germany; 4 Institute of Movement and Neuroscience, German Sport University, Am Sportpark Müngersdorf 6, Cologne, 50933 Germany; 5 Department of Orthopedic Surgery, Sahlgrenska University Hospital, 415 45 Göteborg, Sweden; 6 Centre of Precision Rehabilitation for Spinal Pain (CPR Spine), School of Sport, Exercise and Rehabilitation Sciences, College of Life and Environmental Sciences, University of Birmingham, Edgbaston, B15 2TT UK; 7 NASA Johnson Space Center, 2101 NASA Parkway SD4, Houston, TX 77058 USA; 8 Centre for Military Medicine, Satakunta Air Command, P.O.Box 761, 33101 Tampere, Finland; 9 University of Ulm, Helmholtzstrasse 14, 89081 Ulm, Germany; 10 Orthopaedics and Spine Surgery, Sahlgrenska University Hospital, Bruna Stråket 11B, Göteborg, 413 45 Sweden; 11 Institute of Biomechanics and Orthopaedics, German Sport University Cologne, Am Sportpark Müngersdorf 6, 50933 Cologne, Germany

**Keywords:** Prolapse, Atrophy, Astronaut, Microgravity, Vertebrae, Disc prolapse

## Abstract

**Background:**

Astronauts have a higher risk of cervical intervertebral disc herniation. Several mechanisms have been attributed as causative factors for this increased risk. However, most of the previous studies have examined potential causal factors for lumbar intervertebral disc herniation only. Hence, we aim to conduct a study to identify the various changes in the cervical spine that lead to an increased risk of cervical disc herniation after spaceflight.

**Methods:**

A cohort study with astronauts will be conducted. The data collection will involve four main components: a) Magnetic resonance imaging (MRI); b) cervical 3D kinematics; c) an Integrated Protocol consisting of maximal and submaximal voluntary contractions of the neck muscles, endurance testing of the neck muscles, neck muscle fatigue testing and questionnaires; and d) dual energy X-ray absorptiometry (DXA) examination. Measurements will be conducted at several time points before and after astronauts visit the International Space Station. The main outcomes of interest are adaptations in the cervical discs, muscles and bones.

**Discussion:**

Astronauts are at higher risk of cervical disc herniation, but contributing factors remain unclear. The results of this study will inform future preventive measures for astronauts and will also contribute to the understanding of intervertebral disc herniation risk in the cervical spine for people on Earth. In addition, we anticipate deeper insight into the aetiology of neck pain with this research project.

**Trial registration:**

German Clinical Trials Register, DRKS00026777. Registered on 08 October 2021.

**Supplementary Information:**

The online version contains supplementary material available at 10.1186/s12891-022-05684-0.

## Background

Astronauts have a markedly increased risk of cervical and lumbar intervertebral disc (IVD) herniation after spaceflight [1]. A review of the literature [2] suggested that hyperhydration of the lumbar IVDs during spaceflight is the most probable mechanism for increased lumbar IVD herniation risk post-flight. However, it is not clear whether this is a mechanism occurring within the cervical spine. Further, recent data on the lumbar spine [3, 4] did not reveal increased IVD hydration post-flight, and rather suggested that muscle atrophy may be a potential cause. Hyperhydration of the cervical IVDs may occur during spaceflight, as observed in short-term recumbency [5], but there is no data on this yet.

Beyond IVD hydration, several other factors could influence the injury risk of the cervical spinal tissues, including the discs. For example, muscle function as well as neurovestibular function are known to be impaired after spaceflight, which can impede the control of the neck muscles [6–13]. Data from people with neck pain has revealed that the deep cervical extensors (i.e. suboccipital muscles, m. semispinalis cervicis and multifidus muscle) [14] and deep cervical flexors (i.e. longus colli and capitis) [15] commonly show reduced activation in the presence of pain. However, these muscle groups have not yet been examined in astronauts in any detail. Furthermore, studies [16–18] in patients with neck pain have demonstrated the presence of fat infiltration and pseudohypertrophy of the musculature, which may mask muscle atrophy, and could be an indicator of muscle dysfunction itself.

Further, adaptations of the cervical IVD occurring during spaceflight may be accompanied by changes in the adjacent vertebral bodies and end-plates. The IVD receives its nutrition via the end-plates [19], and end-plate damage is suggested to lead to IVD degeneration [20, 21]. Furthermore, with bone loss of the vertebrae, as in persons with osteoporosis, the (lumbar) IVDs are affected and increase in size [22]. If bone loss occurs in the cervical spine, this may result in deformation of the vertebral end-plate, reduced loads on the disc, and hence could impact disc hydration.

To date, as identified in a prior review [2], there has been no systematic examination of cervical spine adaptations due to weightlessness. Prior ground-based research in prolonged bed-rest [23] indicated that prolonged bed-rest is likely an inadequate model to simulate the effects of spaceflight on the cervical spine.

The aim of this study is therefore to examine potential factors associated with cervical IVD herniations before and after spaceflight. Our a priori hypotheses were that the following will be observed after spaceflight.

- Increases in cervical disc volume
- Increases in cervical disc hydration
- Reduced muscle oxygenation and blood flow in the cervical region
- The deep cervical extensor muscles (i.e. suboccipital muscles, m. semispinalis cervicis and multifidus muscle) and the deep cervical flexors (i.e. m. longus colli and capitis) will undergo atrophy
- An increase in neck muscle fat content
- Reduced range of cervical spine movements
- Reduced speed of cervical spine movements
- Aberrant motions, defined as when the instantaneous helical axis folds back upon itself and movement is not continued anymore, will occur more frequently
- Reduced endurance capacity of the neck muscles
- Decreased neck flexor and extensor maximal strength
- Neck muscle activity will be decreased during maximal and sustained isometric contractions
- Neck flexor-extensor co-activation will increase (during isometric and maximal contractions)
- A more homogenous pattern of spatial motor unit activity
- Increased motor unit discharge rate variability and force tremor (reduced force steadiness)
- Decreased motor unit conduction velocity in the neck muscles
- Cervical spine bone density will decrease
- Increases in cervical disc volume will lead to increased stress and strains in cervical spines during loading upon return to Earth
- Increases in cervical disc volume will lead to increased risk of disc herniations

## Methods

This cohort study will include astronauts who have stayed at the International Space Station (ISS) in order to identify the factors involved in spinal dysfunction and cervical IVD herniation. We will not include pregnant women, subjects that have non-MRI compatible metal implants, or electronic implants. It will be conducted as a multi-center study at the Johnson Space Center, Houston (Texas); European Astronaut Centre, Cologne (Germany) and the University of Texas Medical Branch, Houston (Texas). The planned protocol is in accordance with the Helsinki Declaration of ethical principles [24] and approved by the NASA (Study000000222, NASA MPA Number NASA7116301606HR) and ESA (ILSRA-2014-0033) Institutional Review Board. This study was prospectively registered in the German Clinical Trials Register (Approved on 08 October 2021, registration number DRKS00026777). The STROBE checklist (Supplementary material 1) was used to report the components for this study protocol [25].

The planned investigation will be presented to the crew members by the science team members (DLB, GA) in an Informed Consent Briefing (ICB) of the NASA Human Research Program. Additionally, a video ICB illustrating all required science and operational aspects of the study will be prepared. After the ICB, the crew members have 3–4 weeks to express their interest in participating in the presented study. A written informed consent will be obtained from the participants.

The number of astronauts a research group can recruit for measurements is always limited. Using data from an earlier study [23], we calculated test-retest repeatability of average cervical disc volume (average of all discs from C2/3 to C7/T1) with 1.5 years between testing sessions. We observed a correlation of 0.964 (95% CI: 0.913–0.986) between measures on the same 21 participants separated by 1.5 years. The mean (SD) average cervical disc volume was 2.01(0.31) cm^3^. If 10 astronauts are to be measured, pre- and post-flight, and given the above repeatability data, the calculated detectable effect size would be a 2.07% difference (95%CI: 1.31–3.24%) in average cervical disc volume before and after spaceflight. This assumed a repeated measures ANOVA design and testing for within-factor (i.e. pre-flight vs. post-flight) differences with an alpha-level of 0.05 and a power of 0.80. These calculations were performed in G*Power3 version 3.1.2 [26, 27].

### Session overview

**Fig. 1:**
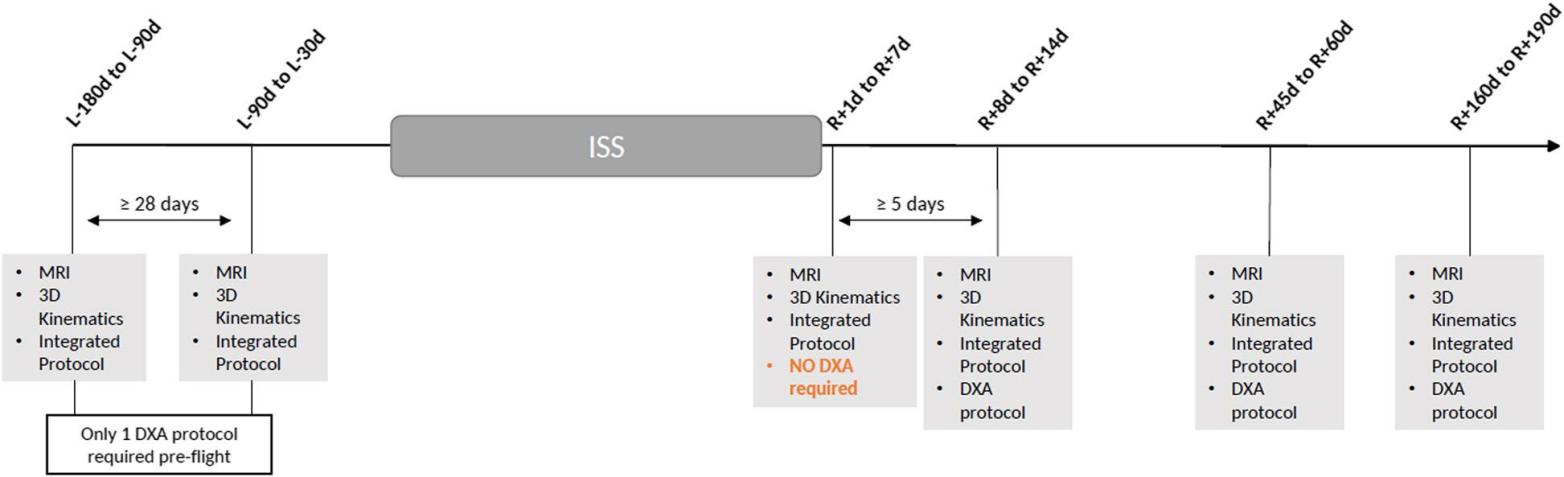
Overview of Measurement Protocol

The study includes only pre−/post-flight sessions comprising different measurements, listed below and illustrated in Fig. 1:

- Magnetic resonance imaging (**MRI**)
- Cervical spine **3D kinematics**
- an **Integrated Protocol** consisting of maximal and submaximal voluntary contractions of the neck muscles, an endurance test of the neck muscles, a neck muscle fatigue test and questionnaires;
- a dual energy X-ray absorptiometry (**DXA**) examination of the cervical spine

In addition to the longitudinal study of the astronauts, it would be desirable to compare the different measures in the astronauts to age and sex matched controls, which could be added as an expansion of the primary study set-up. In this case, the spacing of the tests will be aligned to be a similar duration as per the reference ISS subject. Two subjects on ground will be recruited per ISS subject. If possible, non-flier astronauts shall be recruited.

### Magnetic resonance imaging (MRI)

MRI Measurements will be done for:

- The intervertebral disc and vertebral body morphology and signal characteristics (sagittal T2 relaxography, DIXON and DWI imaging. Scan region: C2 to and including T1)
- Cervical musculature morphology and composition (paraxial: DIXON, T1-weighted imaging. Scan region: base of occiput to C7)

A Siemens Biograph 3 T scanner will be used for the measurements in Cologne, while measurements in Houston will be conducted with a Siemens Magnetom Verio 3 T.

#### Subject position

The subject shall be placed supine with a standard pillow and the head in neutral rotation. To fix the head in the neutral position, foam shall be placed between the head coil and the head of the subject.

### Cervical spine 3D kinematics

**Fig. 2:**
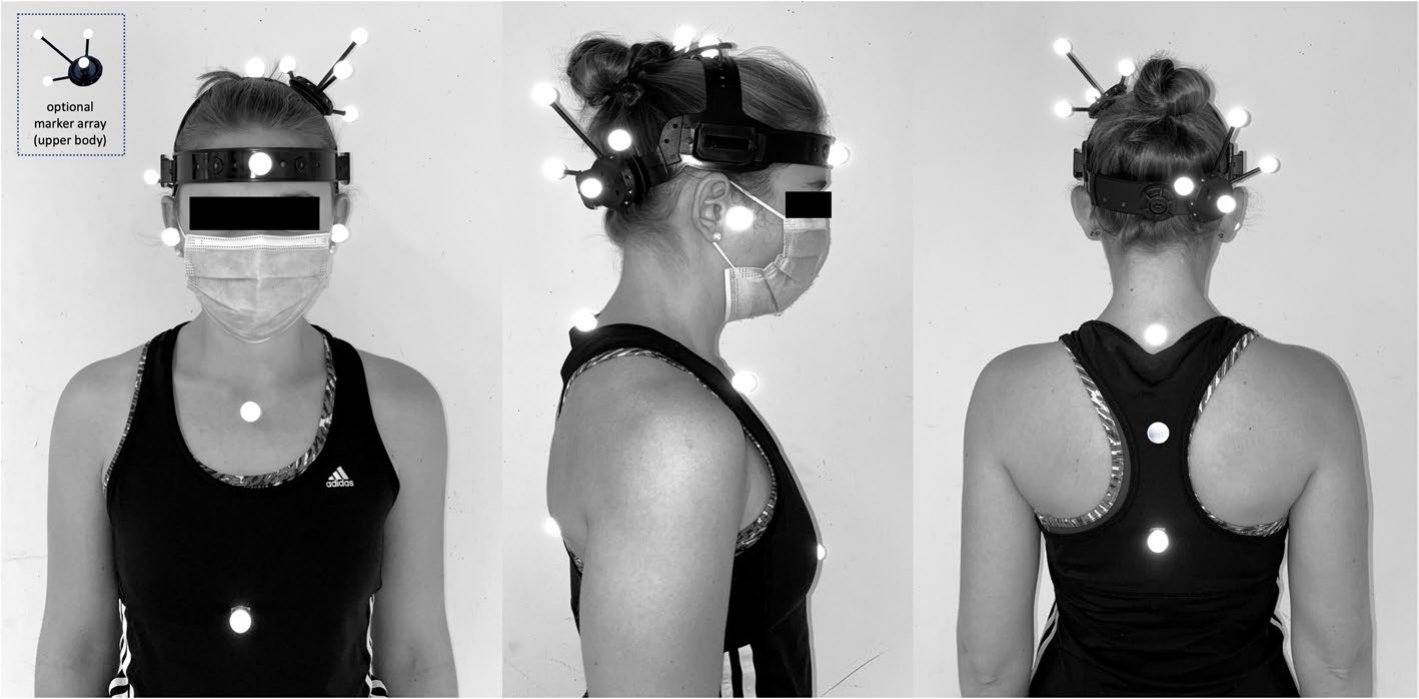
Sample marker set to determine the three-dimensional coordinate system for head and trunk segments and to calculate the helical axis of rotation. Depending on the position of the cameras in the room, the marker set needs to be adjusted and may differ slightly from the image (The images are original pictures taken by the team)

The helical axis approach shall be used to examine aberrations and impairments in cervical spine movement [28–30]. The helical axis model defines a movement as the angle of rotation around a moving axis and provides information about the instantaneous helical axis itself, the instantaneous centre of rotation and the range and speed of the movement. The helical axis shall be determined based on 3D motion capture of reflective markers defining a three-dimensional coordinate system for the head and the trunk [31]. For these measurements, the subject shall be equipped with retroflective markers attached to a headframe and to the trunk (Fig. 2). Depending on the size of the room and the position of the cameras in the room, the marker set may be supplemented by additional technical markers or marker arrays to reconstruct the required anatomical markers.

The subject shall be instructed to perform the neck movements (neck flexion and extension, neck rotation, neck lateral flexion, circumduction) whilst seated, during which the kinematics of the movements are recorded using an infrared-based motion capture system. Three to five repetitions shall be performed for each of the movements in the same order as listed above. Between each movement, a rest period of at least 30 seconds should be provided. In all directions, the subject shall bend as far as possible, under the condition that the movement is painless, with a self-chosen pace. Helical axis descriptors are calculated using the equations described by Spoor and Veldpaus [32].

### Integrated protocol

The following table (Table 1) illustrates the Integrated Protocol in order of execution and respective measurement methods.

**Table 1:** Integrated Protocol Overview

	Variable name	Measurement	2D High-density surface EMG	External force sensor	NIRS (blood flow and O_2_ consumption)	iPad/paper
1	**Clinical examination of muscle force, endurance, neuromuscular control**	Maximal voluntary contractions during: -Neck flexion and extension -shoulder shrug	X	X		
		Submaximal voluntary contractions during: -Neck flexion and extension -shoulder shrug	X	X		
		Endurance test: Sustained bilateral shoulder contraction	X	X		
2	**Neck muscle fatigue test:** **Neck extension**		X	X	X	
3	**Questionnaires**				X	X

### Examination of muscle force, neuromuscular control and endurance

The examination of neck muscle force, endurance and neuromuscular control will consist of maximal voluntary contractions (MVC), brief submaximal voluntary contractions tests, and an endurance test. During these procedures, high density, two-dimensional surface electromyography will be used to assess sternocleidomastoid, splenius capitis and upper trapezius (UT) activity over a large surface area during the contractions. The HDEMG electrodes will be placed only after the 3D Kinematics test is completed because the size of the electrodes would restrain the movement of the participant. Furthermore, a handheld dynamometer (NOD, OT Bioelettronica, Torino, Italy) will be used to measure the participant’s force applied during the different contractions. The OTBioLab+ software (OT Bioelettronica, Torino, Italy) will be used for HDEMG acquisition and to generate the force visual feedback targets for the submaximal contractions. During all tests, the participants will be seated on a stable chair, with their thorax strapped to the chair.

### Electromyography

**Fig. 3:**
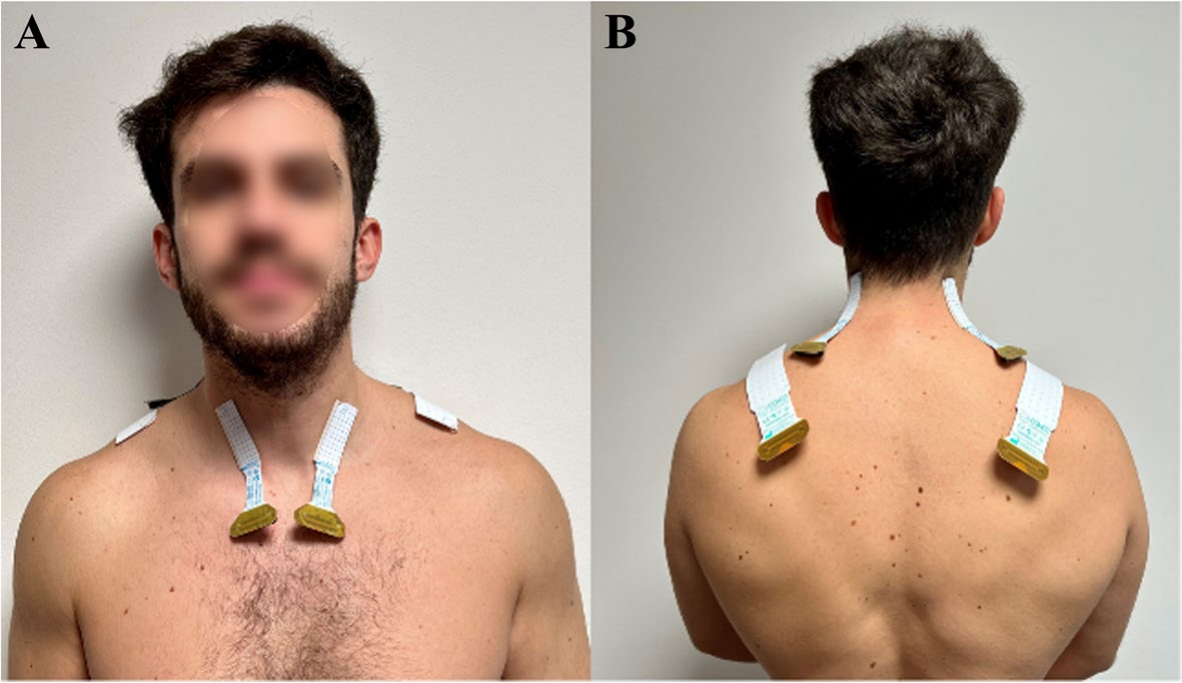
Electrode position over the participant’s **A**) sternocleidomastoid, **B**) splenius capitis and upper trapezius (The images are original pictures taken by the team)

The skin will be prepared prior to electrode placement. This will include shaving, gentle local skin abrasion with abrasive paste (Spes Medica, Italy) to reduce skin impendence and cleaning with water. HDEMG signals will be recorded using six 13 × 5 semi-disposable adhesive matrices of equally spaced electrodes (Fig. 3). The grids placed over the neck muscles will be smaller, with an inter-electrode distance of 4 mm (GR04MM1305, OT Bioelettronica, Torino, Italy), while those placed over the UT will have an interelectrode distance of 8 mm (GR08MM1305, OT Bioelettronica, Torino, Italy). The HDEMG grids will be prepared by attaching a double-side adhesive foam to the electrode (SPES Medica, Genoa, Italy) and the electrode cavities will be filled with a highly conductive-adhesive paste that provides electrode-skin contact (AC-CREAM, SPES Medica, Genoa, Italy). HDEMG signals will be recorded from each muscle bilaterally with the following electrode placement:

- Sternocleidomastoid: Approximately the first 1/3 of the distance between the line from the sternal notch to the mastoid process [33].
- Splenius capitis: From C7 to C2 at the intersection between the sternocleidomastoid and trapezius muscles along the muscle’s line of action (i.e. along a line connecting the top of the ear and C7) [34].
- Upper trapezius: 4th row of the electrode grid in line with C7 and the acromion [35–37].

Reference electrodes (WhiteSensor WS, Ambu A/S, Ballerup, Denmark) will be placed over C7 spinal process and right acromion. A wrist strap (WS1, OT Bioelettronica, Torino, Italy) will be also used as a reference electrode on the left side. All electrodes will be connected to the same bioelectrical amplifier (quattrocento- OT- Bioelettronica, Torino, Italy)

The HDEMG signals will be synchronized with the force signals acquired from the NOD handheld dynamometer (output range: 0-5 V, sampling rate: 100 Hz; Signal gain: 940 V/V) through the auxiliary input of the surface EMG amplifier. All HDEMG signals will be recorded in monopolar mode and a band-pass filter will be applied during the recording (bandwidth: 10–500 Hz, − 3 dB). Both HDEMG and force signals will be amplified by a factor of 150, sampled at 2048 Hz, and digitized with a 16-bit A/D converter through the bioelectrical amplifier.

#### Handheld dynamometer measurements

**Fig. 4:**
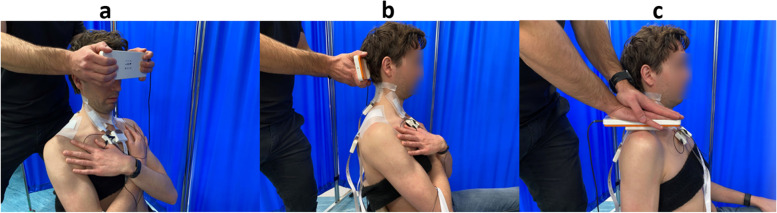
Experimental setup for high-density EMG-force tests. **a** Neck flexion contractions, **b** neck extension contractions and **c** shoulder shrug contractions (The images are original pictures taken by the team)

During the force measurements with the handheld dynamometer, the participants will be asked to perform maximal and submaximal voluntary contractions, with the aim to assess neck muscle strength and neck force control. The participants will perform neck flexion, extension and shoulder shrug. For neck flexion and extension tasks, the handheld dynamometer will be held against the participant’s forehead and occiput respectively (Fig. 4a and b). For the shoulder shrug, the handheld dynamometer will be placed over the participant’s acromion region and the investigator will resist the force exerted by the participant while they shrug their shoulder (Fig. 4c).

##### Neck flexion and extension

During the maximum voluntary isometric contractions (MVC) and submaximal contractions of the neck flexors and extensors, the participant will be asked to cross his/her arms. They will be asked to flex and extend their neck against the resistance of the handheld dynamometer (isometric contraction) and two MVCs will be performed per direction. For the flexion and extension MVCs, the participants will be instructed to push maximally into the dynamometer. Submaximal contractions will be performed at 25 and 50% MVC and will last 20s and 15 s respectively and each contraction level will be performed twice for both flexion and extension. The order of the MVCs (extension/flexion) will be randomized across participants. For example, if the extension is to be tested first, the following procedures will take place: participants will perform one MVC lasting 5 seconds towards extension, rest for 1 minute, and then perform another MVC (5 seconds) in the same direction. The highest MVC will be used to determine the submaximal contraction forces, which will be presented to the participant in a computer monitor placed 60 cm in front of them. Participants will be instructed to match a force target represented as a trapezoid with a force ramp-up and ramp-down phase at a rate of 10%MVC/s, reaching the force target of 25 and 50% MVC. The submaximal contractions at 25 and 50% MVC will last 20s and 15 s respectively. The procedure is as follows: participants will perform two extension contractions at 25% MVC (20 seconds each), with 10s rest between repetitions and then after a 30s rest, another two extension contractions at 50% MVC with 20s rest between repetitions. The order of the submaximal contractions (force level: 25 vs. 50% MVC) will be randomized. The same procedures will be followed for isometric neck flexion contractions.

##### Shoulder shrug

During this measurement, the participant will be sitting on a chair with their arm and forearm extended with their palm facing their body. NOD hand-held dynamometer will be placed over the participant’s acromion region by the investigator who will resist the force exerted by the participant while they shrug their shoulder (Fig. 4c). Then, they will be instructed to shrug their shoulder against the investigator’s resistance. Only the dominant side will be assessed. Similar to the procedure for neck extension/flexion measurements, both MVC and submaximal contractions will be performed. The highest shoulder shrug MVC will be used to determine the shoulder shrug submaximal contraction forces, which will be presented to the participant on the same computer monitor.

#### Endurance test

Fatigability of the UT will be assessed via EMG during a single sustained bilateral shoulder contraction [36]. This test will be performed with the participant seated in the same position as during the previous measurements. The participants will be asked to abduct both arms without any resistance to 90 degrees with their elbows fully extended, their forearms in 90 degrees of pronation, and their palms facing towards the floor for 60s. One aluminium pole will be placed at shoulder level to provide tactile position feedback to the participant.

#### EMG signal processing & analysis

The EMG signal analysis will be performed offline, using a custom script (MATLAB 2020b, The MathWorks Inc., USA). As previously described, [35, 36], the HDEMG signals will be re-referenced offline to form 51 and 59 bipolar channels in the presumed direction of the muscle fibers, for the UT and neck flexors/extensors respectively. The bipolar EMG signals will be bandpass filtered (bandwidth: 20-350 Hz) and the Root Mean Square (RMS) values will be computed for each bipolar channel. Moreover, the average HDEMG amplitude, mean power spectral frequency (MNF) and modified entropy (a measure of the heterogeneity of muscle activity) will be calculated for each muscle [36, 38, 39]. These variables will be calculated for each task and repetition and for all six investigated muscles. The EMG amplitude will be expressed as a percentage of the maximum RMS value obtained during the maximal (baseline) MVC for each muscle. The level of co-activation between the neck flexors and extensors during the contractions will be also quantified as follows: EMGAntagonist/EMGAgonist * 100.

#### Single-motor unit identification

A previously validated algorithm will be also used to decompose the HDEMG signals into individual motor unit spike trains [40] The accuracy of the decomposition will be tested with the silhouette measure, which will be set to ≥0.9 [40]. The signals will be decomposed during the entire duration of the contractions, and the discharge times of the motor units will be transformed into binary spike trains from which all the motor unit firing descriptive statistics (i.e. discharge rate) will be calculated. The mean discharge rate and the discharge rate variability (coefficient of variation for the interspike interval [CoVisi]) will be determined during the stable plateau of force signal. Additionally, motor unit recruitment and de-recruitment thresholds will be defined as the neck flexion/extension/shoulder shrug force (%MVC) at the times when the motor units begin and stop discharging action potentials, respectively. Erroneous discharges will be visually inspected and edited using a custom algorithm [41]. Finally, motor unit activity will be monitored longitudinally [42] which allows tracking of the same motor units across different experimental sessions.

#### Force signal analysis

The force signals will be filtered prior to any calculations with a butterworth filter (bandwidth: 3-15 Hz). The highest peak force exerted during the MVCs (SI: kg) will be used as a measure of maximal neck flexion/extension and shoulder shrug strength for each participant. The absolute peak force values will be then normalised to participants’ body mass (N/kg). The neuromuscular control of all muscles will be assessed by calculating the amplitude of the force fluctuations of the force signal during the submaximal contractions in both absolute terms, as the standard deviation (SD) of the force signal and in relative terms as the coefficient of variation (CoV) of the force signal.

The EMG variables and the force related variables will be calculated for the same time window (i.e., steady part of all contractions), by using a custom-made MATLAB script.

### Neck muscle fatigue test

**Fig. 5:**
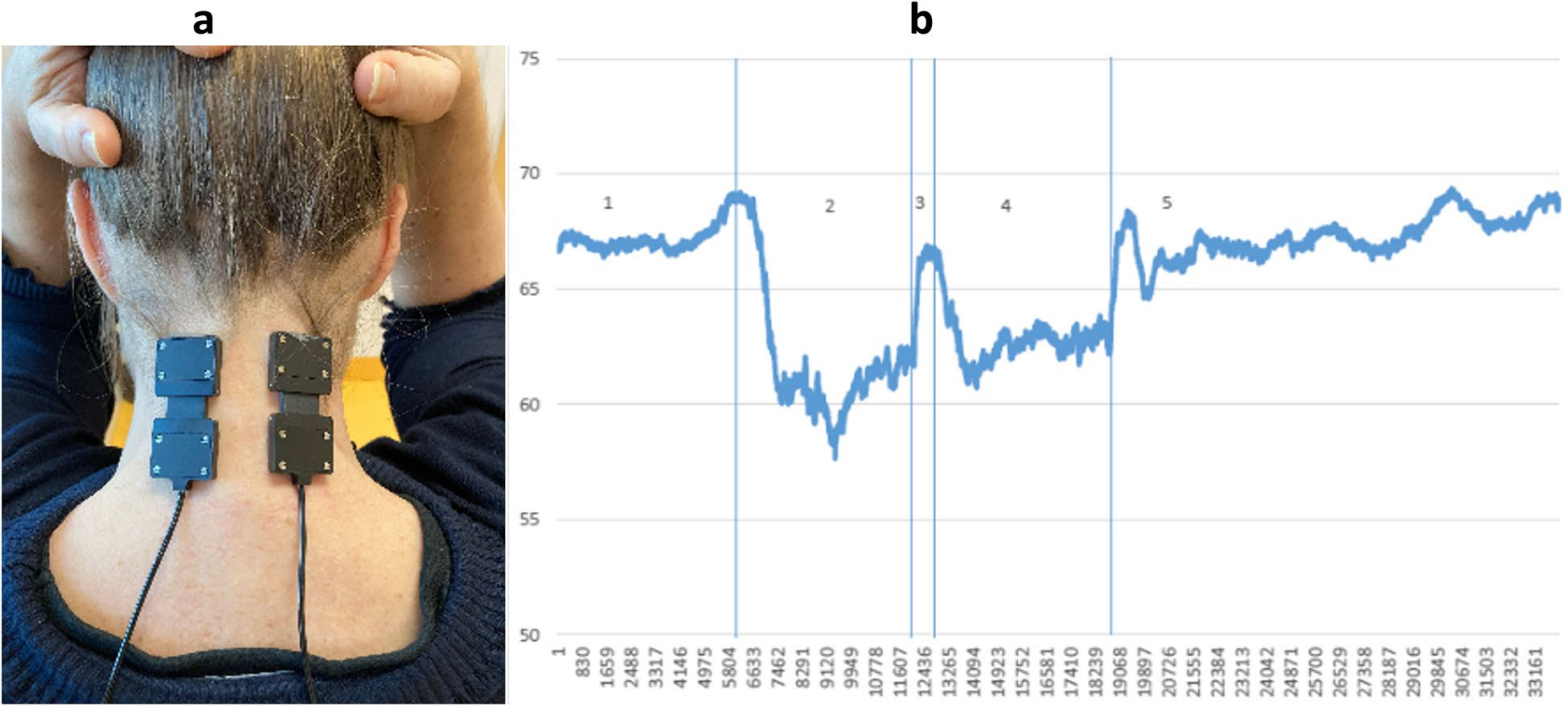
**a** Placement of probes, **b** Image of example data trace. X-axis: time. Y-axis: local muscle oxygen saturation in percent. 1: Baseline values are recorded for 120 sec. 2: 2 minutes extension exercise. 3: Resting 10 seconds. 4: 2 minutes extension, 5: Recovery phase and hyperaemia (The images are original pictures taken by the team)

The Neck Muscle Fatigue Test consists of a sustained neck extension against a submaximal load measured with a handheld dynamometer at the occiput. For this test, the near-infrared spectroscopy (NIRS) will be performed using the PortaLite device (Artinis, Elst, Netherlands) to measure oxygen consumption in the neck skeletal muscles. HDEMG measurements will also be done during the fatigue test for the trapezius muscles only. All the 2D surface EMG grids from the previous tests shall be removed except of the grids placed on the trapezius muscle. The subject will be in sitting position and the NIRS sensors will be attached on the back of the subject’s neck with double-adhesive tape, one on each side on the paravertebral muscles above the level of C7 (Fig. 5a). Dark kinesiotape will be placed over the sensors to avoid artifacts from ambient light. The handheld dynamometer will be positioned at the occiput to monitor subject force output for targeting and feedback purposes. The measurements will be performed continuously before, during and after the neck muscle fatigue test. The NIRS sensors are connected via bluetooth to a laptop which will monitor the local muscle oxygen (StO_2_) in the tissue continuously.

After 2 minutes with recording of the baseline value, the subject shall perform a neck muscle fatigue test, i.e. the participants shall extend isometrically the neck from neutral position against a submaximal load. If the participant feels fatigued but latest after 2 minutes, a resting period of 10 seconds is implemented and monitoring continued. The procedure shall be repeated one more time afterwards. After the second extension task, a 2 minutes resting period will be recorded (Fig. 5b).

### Questionnaires

Questionnaires will be used to assess factors related to cervical spine history, neck pain, prior aviator experience and physical activity (Supplementary Material 2). They will be applied electronically with an iPad (Apple Inc., USA) and/or in paper.

### DXA protocol

**Fig. 6:**
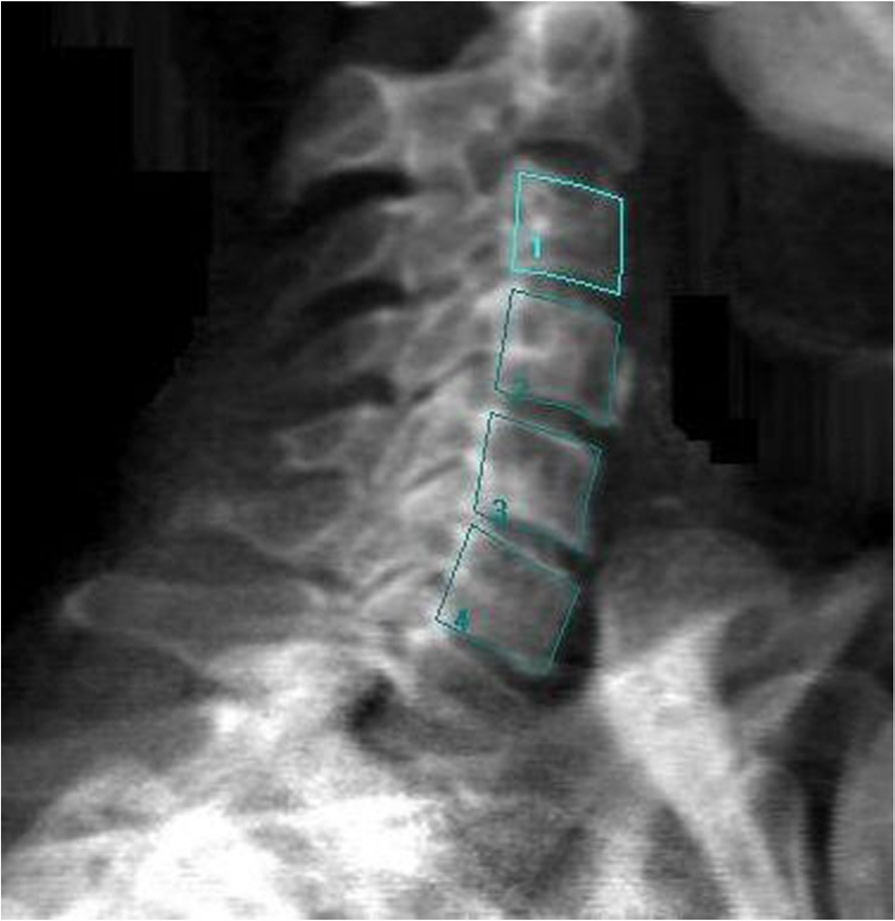
Example image of DXA cervical spine scan with regions of interest traced. Note that “1”, “2”, “3”, “4” refer, respectively to C3, C4, C5 and C6

The measurement of the cervical spine will be performed on a standard DXA machine using the lateral spine setting. The DXA scan will start at the level of T1 and will continue until C1 (Fig. 6). For DXA devices without C-arm, the subject shall be placed in lateral position; for devices with C-arm, the subject shall be placed in supine position with the cervical spine in one line with the thoracic spine to avoid rotation artifacts. A stable foam pillow shall be put under the head to get the cervical spine parallel to the table. Both shoulders will be drawn as far as possible forward (in lateral position only) and downward to prevent a superimposition of the shoulder with the cervical spine. The subject shall be instructed to remain still during scanning (as scans might need to be repeated).

The evaluation will be performed manually with creation of custom (instead of “standard”) ROIs individually for the vertebral bodies C3 to C6 for the respective subject. Subject and vertebrae specific ROIs will be saved as an individual ROI template and used for evaluation of follow-up measurements to ensure reproducibility.

### Blinding and bias control

To reduce the risk of bias, we will assign random codes to each measurement timepoint for each participant in the study. Thus, the individual performing the data analysis will not be aware of the individual participants. Also, the EMG measurement order will be randomized and instructions to the participants will be standardized to reduce the risk of bias.

### Statistical analysis

We will use the R software (× 64 v4.0.2) for statistical analysis. A repeated measures ANOVA design and testing for within-factor (i.e. pre-flight vs. post-flight) is planned for each parameter. If assumptions of normality of distribution of residuals are not held (as assessed via quantile-quantile plots), then non-parametric methods for longitudinal data [43] will be implemented. Where some data is missing, a maximum likelihood based approach will be used [44]. Due to the large number of statistical comparisons to be performed as part of hypothesis testing, the false discovery rate method will be used to reduce the risk of type I statistical error. We will use a significance level of *p* < 0.05.

### Further data analysis, 3D modelling, laboratory testing

Using the data obtained from testing with the astronaut collective, the following laboratory examinations will be performed:

- Cervical IVDs will be loaded in a dynamic disc-loading simulator [45] to provoke IVD herniation and lesions. Three groups of specimens and the parameters adjusted pending outcomes from the astronaut testing: (i) normal discs, (ii) hyperhydrated discs after storing the disc-segments in hypo-osmotic fluid (lower osmolarity), and (iii) hypohydrated discs after storing the disc-segments in hyper-osmotic fluid (high osmolarity). A load combination with flexion, lateral bending right, axial rotation right, and axial compression can reproducibly create a posterior or postero-lateral herniation. The herniations and lesions will be investigated with Ultra-High Field 11.7 Tesla MRI [46].
- FEM studies: calculations of stresses and strains with the IVD in different hydration states and complex motions will be performed.

## Discussion

The results of this study will have broader implications for understanding intervertebral disc herniation risk in the cervical spine in the wider population on Earth. At a later stage, this will also help in developing proper countermeasures to minimize the risk of developing such conditions in astronauts. In case the drop-out rate is higher than expected, the project will take more time since the number of astronauts is limited.

The data from this project will also be used as an input for ground-based in-vitro studies with cadaver spines in a custom-built dynamic disc loading simulator to provoke disc herniations in normal and hydrated discs. Additionally, it will be used as an input to ground-based finite element modelling to estimate stress and strains in discs at different locations during motion, both for normal and hyperhydrated discs.

This includes several different investigations on the cervical spine to capture a range of possible influencing factors. The potential limitations of the current project is the lack of a non-astronaut ground-based control collected. This would help to control for the effect of normal aging in the findings. In our original peer-reviewed research proposal, we planned a 2:1 ratio for time-synchronous ground-based controls to astronauts, but we have not yet secured funding to support this. The generalisability of the findings will be limited to astronauts.

## Supplementary Information

**Additional file 1.**

**Additional file 2.**

**Additional file 3.**

## Data Availability

Not applicable, no data reported in this protocol manuscript. All data arising from the project will be shared in compliance with the ESA Policy on Personal Data Protection PDP. The ESA Data Protection Officer can be contacted on dpo@esa.int.

## Abbreviations

3 T: 3 Tesla
ANOVA: Analysis of variance
CI: Confidence interval
DWI: Diffusion-weighted image
DXA: Dual energy X-ray absorptiometry
ESA: European Space Agency
FEM: Finite element method
HDEMG: High-density electromyography
ICB: Informed Consent Briefing
ILSRA: International Life Sciences Research Announcement
ISS: International Space Station
IVD: Intervertebral disc
MNF: Mean power spectral frequency
MPA: Mentor-Protégé Agreement
MRI: Magnetic resonance imaging
MVC: Maximal voluntary contractions
NASA: National Aeronautics and Space Administration
NIRS: Near-infrared spectroscopy
RMS: Root Mean Square
SD: Standard deviation
UT: Upper trapezius
