# Supplementary material for: Cervical spine and muscle adaptation after spaceflight and relationship to herniation risk: protocol from ‘Cervical in Space’ trial
Source: BMC Musculoskelet Disord. 2022 Aug 13;23:772. doi: 10.1186/s12891-022-05684-0 (PMC9375326; doi:10.1186/s12891-022-05684-0)
Supplement: Supplementary file 2 — Additional file 2. [file 12891_2022_5684_MOESM2_ESM.docx]

**QUESTIONNAIRE FOR THE FIRST PRE-FLIGHT SESSION**

**DEMOGRAPHICS**

Age: Please indicate your age: _____

Gender:

| Male: |  | Female: |  |
| --- | --- | --- | --- |

Height: _____ centimeters

Weight: _____ kg

**FLIGHT EXPERIENCE, AIRCRAFT TYPE & PAST SPACEFLIGHT EXPERIENCE**

Please indicate (with X) your possible previous aircrew career:

- Military ____
- Civilian ____
- No flying background ____

In which year (yyyy) did you begin your flying career? ____

From your logbook, what is your total number of flying hours to date? _____

Please specify the type of aircraft you have been flying, and include the total numbers of hours logged on that type:

| Aircraft Type | Total hours |
| --- | --- |
| High performance jets |  |
| Helicopters |  |
| Transport / Airliner |  |
| Other: |  |
| Other: |  |

Space flight experience

How many space flights you have flown? _______________________________

How many days you have been in space? ________________________________

What is the total hours you have logged in EVA? ____________________________

**PHYSICAL ACTIVITY**

Please indicate, on average, how often you have exercised over the previous year and what form of exercise you have undertaken?

|  | Every Day | 2-5 x / week | 1 x / week | 1-3 x / month | Less than 1 x / month | never |
| --- | --- | --- | --- | --- | --- | --- |
| Running or jogging |  |  |  |  |  |  |
| Cycling (including ergometer) |  |  |  |  |  |  |
| Swimming |  |  |  |  |  |  |
| Rowing (including ergometer) |  |  |  |  |  |  |
| Resistive exercises |  |  |  |  |  |  |
| Core stability training |  |  |  |  |  |  |
| Specific neck exercises |  |  |  |  |  |  |
| Ball games (basketball, tennis, soccer, hockey, etc…) |  |  |  |  |  |  |
| Other (specify; auto generate additional rows as needed) |  |  |  |  |  |  |

**DIET QUESTIONS**

How many cups of caffeinated coffee did you consume in last 24 hours:

How many cups of caffeinated tea did you consume in last 24 hours:

How many cups of caffeinated cola did you consume in last 24 hours:

(Conversions to be done as per this publication https://academic.oup.com/aje/article/146/1/32/81667)

How many glasses of beer did you consume in last 24 hours:

How many glasses of wine did you consume in last 24 hours:

How many glasses of spirits did you consume in last 24 hours:

(Conversions to be done as per this publication https://academic.oup.com/aje/article/146/1/32/81667)

**PAST SYMPTOMS**

Have you ever had any:

- neck pain? [no/yes: auto display additional fields below]

- arm pain, pins and needles or numbness? [no/yes: auto display additional fields below]

- shoulder pain? [no/yes: auto display additional fields below]

- headaches? [no/yes: auto display additional fields below]

If yes to any, for each:

- How long did it last for (in days)?
- Did you receive treatment? What kind?
- Was a diagnosis made? If "yes", what was this?

**CURRENT SYMPTOMS**

Do you currently have any shoulder or arm pain? If yes:

- Please mark the intensity of the pain on this scale [present visual analogue scale to astronaut]
- How did it start (e.g. movement, injury, prolonged sitting, or waking in morning with pain)? [free text entry]
- Is there anything that makes it better? [free text entry]
- Is there anything that makes it worse? [free text entry]
- Do you experience any pins and needles or numbness in your arm? [yes, no answer]
  - If “yes”: describe what parts of your arm are affected

Do you currently have a headache? If yes:

- Please mark the intensity of the pain on this scale [present visual analogue scale to astronaut]
- How did it start (e.g. movement, injury, prolonged sitting, or waking in morning with pain)? [free text entry]
- Is there anything that makes it better? [free text entry]
- Is there anything that makes it worse? [free text entry]

Do you currently have any neck pain? If yes:

- Please mark the intensity of the pain on this scale [present visual analogue scale to astronaut]
- How did it start (e.g. movement, injury, prolonged sitting, or waking in morning with pain)? [free text entry]
- Is there anything that makes it better? [free text entry]
- Is there anything that makes it worse? [free text entry]
- Do you experience any pins and needles or numbness in your shoulder or arm? [yes, no answer]

If the astronaut reports current NECK pain, display the NDI:

Pain Intensity

- I have no pain at the moment
- The pain is very mild at the moment
- The pain is moderate at the moment
- The pain is fairly severe at the moment
- The pain is very severe at the moment
- The pain is the worst imaginable at the moment

Personal Care (Washing, Dressing, etc.)

- I can look after myself normally without causing extra pain
- I can look after myself normally but it causes extra pain
- It is painful to look after myself and I am slow and careful
- I need some help but can manage most of my personal care
- I need help every day in most aspects of self care
- I do not get dressed, I wash with difficulty and stay in bed

Lifting

- I can lift heavy weights without extra pain
- I can lift heavy weights but it gives extra pain
- Pain prevents me lifting heavy weights off the floor, but I can manage if they are conveniently placed, for example on a table
- Pain prevents me from lifting heavy weights but I can manage light to medium weights if they are conveniently positioned
- I can only lift very light weights
- I cannot lift or carry anything

Reading

- I can read as much as I want to with no pain in my neck
- I can read as much as I want to with slight pain in my neck
- I can read as much as I want with moderate pain in my neck
- I can’t read as much as I want because of moderate pain in my neck
- I can hardly read at all because of severe pain in my neck
- I cannot read at all

Headaches

- I have no headaches at all
- I have slight headaches, which come infrequently
- I have moderate headaches, which come infrequently
- I have moderate headaches, which come frequently
- I have severe headaches, which come frequently
- I have headaches almost all the time

Concentration

- I can concentrate fully when I want to with no difficulty
- I can concentrate fully when I want to with slight difficulty
- I have a fair degree of difficulty in concentrating when I want to
- I have a lot of difficulty in concentrating when I want to
- I have a great deal of difficulty in concentrating when I want to
- I cannot concentrate at all

Work

- I can do as much work as I want to
- I can only do my usual work, but no more
- I can do most of my usual work, but no more
- I cannot do my usual work
- I can hardly do any work at all
- I can’t do any work at all

Driving

- I can drive my car without any neck pain
- I can drive my car as long as I want with slight pain in my neck
- I can drive my car as long as I want with moderate pain in my neck
- I can’t drive my car as long as I want because of moderate pain in my neck
- I can hardly drive at all because of severe pain in my neck
- I can’t drive my car at all

Sleeping

- I have no trouble sleeping
- My sleep is slightly disturbed (less than 1 hr sleepless)
- My sleep is mildly disturbed (1-2 hrs sleepless)
- My sleep is moderately disturbed (2-3 hrs sleepless)
- My sleep is greatly disturbed (3-5 hrs sleepless)
- My sleep is completely disturbed (5-7 hrs sleepless)

Recreation

- I am able to engage in all my recreation activities with no neck pain at all
- I am able to engage in all my recreation activities, with some pain in my neck
- I am able to engage in most, but not all of my usual recreation activities because of

pain in my neck

- I am able to engage in a few of my usual recreation activities because of pain in

my neck

- I can hardly do any recreation activities because of pain in my neck

I can’t do any recreation activities at all

FREE TEXT ENTRY BOX FOR THE EXAMINER: Examiner to catalogue any further relevant information. I.e. more detailed subjective examination where pain/parathesia is present.

**QUESTIONNAIRE FOR SECOND PRE-FLIGHT SESSION**

**SYMPTOMS SINCE LAST SESSION**

Since your last testing session for this experiment, have you had any:

- neck pain? [no/yes: auto display additional fields below]

- shoulder pain? [no/yes: auto display additional fields below]

- arm pain, pins and needles or numbness? [no/yes: auto display additional fields below]

- headaches [no/yes: auto display additional fields below]

If yes to any, for each:

- How long did the pain last for (in days)?
- Did you receive treatment? What kind?
- Was a diagnosis made? If "yes", what was this?

**PHYSICAL ACTIVITY**

Please indicate, on average, how often you currently exercise:

|  | Every Day | 2-5 x / week | 1 x / week | 1-3 x / month | Less than 1 x / month | never |
| --- | --- | --- | --- | --- | --- | --- |
| Running or jogging |  |  |  |  |  |  |
| Cycling (including ergometer) |  |  |  |  |  |  |
| Swimming |  |  |  |  |  |  |
| Rowing (including ergometer) |  |  |  |  |  |  |
| Resistive exercises |  |  |  |  |  |  |
| Core stability training |  |  |  |  |  |  |
| Specific neck exercises |  |  |  |  |  |  |
| Ball games (basketball, tennis, soccer, hockey, etc…) |  |  |  |  |  |  |
| Other (specify; auto generate additional rows as needed) |  |  |  |  |  |  |

**DIET QUESTIONS**

How many cups of caffeinated coffee did you consume in last 24 hours:

How many cups of caffeinated tea did you consume in last 24 hours:

How many cups of caffeinated cola did you consume in last 24 hours:

(Conversions to be done as per this publication https://academic.oup.com/aje/article/146/1/32/81667)

How many glasses of beer did you consume in last 24 hours:

How many glasses of wine did you consume in last 24 hours:

How many glasses of spirits did you consume in last 24 hours:

(Conversions to be done as per this publication https://academic.oup.com/aje/article/146/1/32/81667)

**CURRENT SYMPTOMS**

Do you currently have any shoulder or arm pain? If yes:

- Please mark the intensity of the pain on this scale [present visual analogue scale to astronaut]
- How did it start (e.g. movement, injury, prolonged sitting, or waking in morning with pain)? [free text entry]
- Is there anything that makes it better? [free text entry]
- Is there anything that makes it worse? [free text entry]
- Do you experience any pins and needles or numbness in your arm? [yes, no answer]
  - If “yes”: describe what parts of your arm are affected

Do you currently have a headache? If yes:

- Please mark the intensity of the pain on this scale [present visual analogue scale to astronaut]
- How did it start (e.g. movement, injury, prolonged sitting, or waking in morning with pain)? [free text entry]
- Is there anything that makes it better? [free text entry]
- Is there anything that makes it worse? [free text entry]

Do you currently have any neck pain? If yes:

- Please mark the intensity of the pain on this scale [present visual analogue scale to astronaut]
- How did it start (e.g. movement, injury, prolonged sitting, or waking in morning with pain)? [free text entry]
- Is there anything that makes it better? [free text entry]
- Is there anything that makes it worse? [free text entry]
- Do you experience any pins and needles or numbness in your shoulder or arm? [yes, no answer]

If the astronaut reports current NECK pain, display the NDI:

Pain Intensity

- I have no pain at the moment
- The pain is very mild at the moment
- The pain is moderate at the moment
- The pain is fairly severe at the moment
- The pain is very severe at the moment
- The pain is the worst imaginable at the moment

Personal Care (Washing, Dressing, etc.)

- I can look after myself normally without causing extra pain
- I can look after myself normally but it causes extra pain
- It is painful to look after myself and I am slow and careful
- I need some help but can manage most of my personal care
- I need help every day in most aspects of self care
- I do not get dressed, I wash with difficulty and stay in bed

Lifting

- I can lift heavy weights without extra pain
- I can lift heavy weights but it gives extra pain
- Pain prevents me lifting heavy weights off the floor, but I can manage if they are conveniently placed, for example on a table
- Pain prevents me from lifting heavy weights but I can manage light to medium weights if they are conveniently positioned
- I can only lift very light weights
- I cannot lift or carry anything

Reading

- I can read as much as I want to with no pain in my neck
- I can read as much as I want to with slight pain in my neck
- I can read as much as I want with moderate pain in my neck
- I can’t read as much as I want because of moderate pain in my neck
- I can hardly read at all because of severe pain in my neck
- I cannot read at all

Headaches

- I have no headaches at all
- I have slight headaches, which come infrequently
- I have moderate headaches, which come infrequently
- I have moderate headaches, which come frequently
- I have severe headaches, which come frequently
- I have headaches almost all the time

Concentration

- I can concentrate fully when I want to with no difficulty
- I can concentrate fully when I want to with slight difficulty
- I have a fair degree of difficulty in concentrating when I want to
- I have a lot of difficulty in concentrating when I want to
- I have a great deal of difficulty in concentrating when I want to
- I cannot concentrate at all

Work

- I can do as much work as I want to
- I can only do my usual work, but no more
- I can do most of my usual work, but no more
- I cannot do my usual work
- I can hardly do any work at all
- I can’t do any work at all

Driving

- I can drive my car without any neck pain
- I can drive my car as long as I want with slight pain in my neck
- I can drive my car as long as I want with moderate pain in my neck
- I can’t drive my car as long as I want because of moderate pain in my neck
- I can hardly drive at all because of severe pain in my neck
- I can’t drive my car at all

Sleeping

- I have no trouble sleeping
- My sleep is slightly disturbed (less than 1 hr sleepless)
- My sleep is mildly disturbed (1-2 hrs sleepless)
- My sleep is moderately disturbed (2-3 hrs sleepless)
- My sleep is greatly disturbed (3-5 hrs sleepless)
- My sleep is completely disturbed (5-7 hrs sleepless)

Recreation

- I am able to engage in all my recreation activities with no neck pain at all
- I am able to engage in all my recreation activities, with some pain in my neck
- I am able to engage in most, but not all of my usual recreation activities because of

pain in my neck

- I am able to engage in a few of my usual recreation activities because of pain in

my neck

- I can hardly do any recreation activities because of pain in my neck
- I can’t do any recreation activities at all

FREE TEXT ENTRY BOX FOR THE EXAMINER: Examiner to catalogue any further relevant information. I.e. more detailed subjective examination where pain/parathesia is present.

**QUESTIONNAIRE FOR FIRST POST-FLIGHT SESSION**

**SPACEFLIGHT**

How many days was your spaceflight?

[Autogenerate a scale for astronaut to mark flight days where had pain]

If you had any neck pain during flight, please mark on what flight days (approximately) this was:

If you had any shoulder pain during flight, please mark on what flight days (approximately) this was:

If you had any headaches pain during flight, please mark on what flight days (approximately) this was:

**DIET QUESTIONS**

How many cups of caffeinated coffee did you consume in last 24 hours:

How many cups of caffeinated tea did you consume in last 24 hours:

How many cups of caffeinated cola did you consume in last 24 hours:

(Conversions to be done as per this publication https://academic.oup.com/aje/article/146/1/32/81667)

How many glasses of beer did you consume in last 24 hours:

How many glasses of wine did you consume in last 24 hours:

How many glasses of spirits did you consume in last 24 hours:

(Conversions to be done as per this publication https://academic.oup.com/aje/article/146/1/32/81667)

**CURRENT SYMPTOMS**

Do you currently have any shoulder or arm pain? If yes:

- Please mark the intensity of the pain on this scale [present visual analogue scale to astronaut]
- How did it start (e.g. movement, injury, prolonged sitting, or waking in morning with pain)? [free text entry]
- Is there anything that makes it better? [free text entry]
- Is there anything that makes it worse? [free text entry]
- Do you experience any pins and needles or numbness in your arm? [yes, no answer]
  - If “yes”: describe what parts of your arm are affected

Do you currently have a headache? If yes:

- Please mark the intensity of the pain on this scale [present visual analogue scale to astronaut]
- How did it start (e.g. movement, injury, prolonged sitting, or waking in morning with pain)? [free text entry]
- Is there anything that makes it better? [free text entry]
- Is there anything that makes it worse? [free text entry]

Do you currently have any neck pain? If yes:

- Please mark the intensity of the pain on this scale [present visual analogue scale to astronaut]
- How did it start (e.g. movement, injury, prolonged sitting, or waking in morning with pain)? [free text entry]
- Is there anything that makes it better? [free text entry]
- Is there anything that makes it worse? [free text entry]
- Do you experience any pins and needles or numbness in your shoulder or arm? [yes, no answer]

If the astronaut reports current NECK pain, display the NDI:

Pain Intensity

- I have no pain at the moment
- The pain is very mild at the moment
- The pain is moderate at the moment
- The pain is fairly severe at the moment
- The pain is very severe at the moment
- The pain is the worst imaginable at the moment

Personal Care (Washing, Dressing, etc.)

- I can look after myself normally without causing extra pain
- I can look after myself normally but it causes extra pain
- It is painful to look after myself and I am slow and careful
- I need some help but can manage most of my personal care
- I need help every day in most aspects of self care
- I do not get dressed, I wash with difficulty and stay in bed

Lifting

- I can lift heavy weights without extra pain
- I can lift heavy weights but it gives extra pain
- Pain prevents me lifting heavy weights off the floor, but I can manage if they are conveniently placed, for example on a table
- Pain prevents me from lifting heavy weights but I can manage light to medium weights if they are conveniently positioned
- I can only lift very light weights
- I cannot lift or carry anything

Reading

- I can read as much as I want to with no pain in my neck
- I can read as much as I want to with slight pain in my neck
- I can read as much as I want with moderate pain in my neck
- I can’t read as much as I want because of moderate pain in my neck
- I can hardly read at all because of severe pain in my neck
- I cannot read at all

Headaches

- I have no headaches at all
- I have slight headaches, which come infrequently
- I have moderate headaches, which come infrequently
- I have moderate headaches, which come frequently
- I have severe headaches, which come frequently
- I have headaches almost all the time

Concentration

- I can concentrate fully when I want to with no difficulty
- I can concentrate fully when I want to with slight difficulty
- I have a fair degree of difficulty in concentrating when I want to
- I have a lot of difficulty in concentrating when I want to
- I have a great deal of difficulty in concentrating when I want to
- I cannot concentrate at all

Work

- I can do as much work as I want to
- I can only do my usual work, but no more
- I can do most of my usual work, but no more
- I cannot do my usual work
- I can hardly do any work at all
- I can’t do any work at all

Driving

- I can drive my car without any neck pain
- I can drive my car as long as I want with slight pain in my neck
- I can drive my car as long as I want with moderate pain in my neck
- I can’t drive my car as long as I want because of moderate pain in my neck
- I can hardly drive at all because of severe pain in my neck
- I can’t drive my car at all

Sleeping

- I have no trouble sleeping
- My sleep is slightly disturbed (less than 1 hr sleepless)
- My sleep is mildly disturbed (1-2 hrs sleepless)
- My sleep is moderately disturbed (2-3 hrs sleepless)
- My sleep is greatly disturbed (3-5 hrs sleepless)
- My sleep is completely disturbed (5-7 hrs sleepless)

Recreation

- I am able to engage in all my recreation activities with no neck pain at all
- I am able to engage in all my recreation activities, with some pain in my neck
- I am able to engage in most, but not all of my usual recreation activities because of

pain in my neck

- I am able to engage in a few of my usual recreation activities because of pain in

my neck

- I can hardly do any recreation activities because of pain in my neck

I can’t do any recreation activities at all

**PHYSICAL ACTIVITY**

Please indicate, on average, how often you currently exercise:

|  | Every Day | 2-5 x / week | 1 x / week | 1-3 x / month | Less than 1 x / month | never |
| --- | --- | --- | --- | --- | --- | --- |
| Running or jogging |  |  |  |  |  |  |
| Cycling (including ergometer) |  |  |  |  |  |  |
| Swimming |  |  |  |  |  |  |
| Rowing (including ergometer) |  |  |  |  |  |  |
| Resistive exercises |  |  |  |  |  |  |
| Core stability training |  |  |  |  |  |  |
| Specific neck exercises |  |  |  |  |  |  |
| Ball games (basketball, tennis, soccer, hockey, etc…) |  |  |  |  |  |  |
| Other (specify; auto generate additional rows as needed) |  |  |  |  |  |  |

FREE TEXT ENTRY BOX FOR THE EXAMINER: Examiner to catalogue any further relevant information. I.e. more detailed subjective examination where pain/parathesia is present.

**SUBSEQUENT POST-FLIGHT TESTING SESSIONS**

**DIET QUESTIONS**

How many cups of caffeinated coffee did you consume in last 24 hours:

How many cups of caffeinated tea did you consume in last 24 hours:

How many cups of caffeinated cola did you consume in last 24 hours:

(Conversions to be done as per this publication https://academic.oup.com/aje/article/146/1/32/81667)

How many glasses of beer did you consume in last 24 hours:

How many glasses of wine did you consume in last 24 hours:

How many glasses of spirits did you consume in last 24 hours:

(Conversions to be done as per this publication https://academic.oup.com/aje/article/146/1/32/81667)

**SYMPTOMS SINCE LAST SESSION**

Since your last testing session for this experiment, have you had any:

- neck pain? [no/yes: auto display additional fields below]

- shoulder pain? [no/yes: auto display additional fields below]

- arm pain, pins and needles or numbness? [no/yes: auto display additional fields below]

- headaches [no/yes: auto display additional fields below]

If yes to any, for each:

- How long did the pain last for (in days)?
- Did you receive treatment? What kind?
- Was a diagnosis made? If "yes", what was this?

**CURRENT SYMPTOMS**

Do you currently have any shoulder or arm pain? If yes:

- Please mark the intensity of the pain on this scale [present visual analogue scale to astronaut]
- How did it start (e.g. movement, injury, prolonged sitting, or waking in morning with pain)? [free text entry]
- Is there anything that makes it better? [free text entry]
- Is there anything that makes it worse? [free text entry]
- Do you experience any pins and needles or numbness in your arm? [yes, no answer]
  - If “yes”: describe what parts of your arm are affected

Do you currently have a headache? If yes:

- Please mark the intensity of the pain on this scale [present visual analogue scale to astronaut]
- How did it start (e.g. movement, injury, prolonged sitting, or waking in morning with pain)? [free text entry]
- Is there anything that makes it better? [free text entry]
- Is there anything that makes it worse? [free text entry]

Do you currently have any neck pain? If yes:

- Please mark the intensity of the pain on this scale [present visual analogue scale to astronaut]
- How did it start (e.g. movement, injury, prolonged sitting, or waking in morning with pain)? [free text entry]
- Is there anything that makes it better? [free text entry]
- Is there anything that makes it worse? [free text entry]
- Do you experience any pins and needles or numbness in your shoulder or arm? [yes, no answer]

If the astronaut reports current NECK pain, display the NDI:

Pain Intensity

- I have no pain at the moment
- The pain is very mild at the moment
- The pain is moderate at the moment
- The pain is fairly severe at the moment
- The pain is very severe at the moment
- The pain is the worst imaginable at the moment

Personal Care (Washing, Dressing, etc.)

- I can look after myself normally without causing extra pain
- I can look after myself normally but it causes extra pain
- It is painful to look after myself and I am slow and careful
- I need some help but can manage most of my personal care
- I need help every day in most aspects of self care
- I do not get dressed, I wash with difficulty and stay in bed

Lifting

- I can lift heavy weights without extra pain
- I can lift heavy weights but it gives extra pain
- Pain prevents me lifting heavy weights off the floor, but I can manage if they are conveniently placed, for example on a table
- Pain prevents me from lifting heavy weights but I can manage light to medium weights if they are conveniently positioned
- I can only lift very light weights
- I cannot lift or carry anything

Reading

- I can read as much as I want to with no pain in my neck
- I can read as much as I want to with slight pain in my neck
- I can read as much as I want with moderate pain in my neck
- I can’t read as much as I want because of moderate pain in my neck
- I can hardly read at all because of severe pain in my neck
- I cannot read at all

Headaches

- I have no headaches at all
- I have slight headaches, which come infrequently
- I have moderate headaches, which come infrequently
- I have moderate headaches, which come frequently
- I have severe headaches, which come frequently
- I have headaches almost all the time

Concentration

- I can concentrate fully when I want to with no difficulty
- I can concentrate fully when I want to with slight difficulty
- I have a fair degree of difficulty in concentrating when I want to
- I have a lot of difficulty in concentrating when I want to
- I have a great deal of difficulty in concentrating when I want to
- I cannot concentrate at all

Work

- I can do as much work as I want to
- I can only do my usual work, but no more
- I can do most of my usual work, but no more
- I cannot do my usual work
- I can hardly do any work at all
- I can’t do any work at all

Driving

- I can drive my car without any neck pain
- I can drive my car as long as I want with slight pain in my neck
- I can drive my car as long as I want with moderate pain in my neck
- I can’t drive my car as long as I want because of moderate pain in my neck
- I can hardly drive at all because of severe pain in my neck
- I can’t drive my car at all

Sleeping

- I have no trouble sleeping
- My sleep is slightly disturbed (less than 1 hr sleepless)
- My sleep is mildly disturbed (1-2 hrs sleepless)
- My sleep is moderately disturbed (2-3 hrs sleepless)
- My sleep is greatly disturbed (3-5 hrs sleepless)
- My sleep is completely disturbed (5-7 hrs sleepless)

Recreation

- I am able to engage in all my recreation activities with no neck pain at all
- I am able to engage in all my recreation activities, with some pain in my neck
- I am able to engage in most, but not all of my usual recreation activities because of

pain in my neck

- I am able to engage in a few of my usual recreation activities because of pain in

my neck

- I can hardly do any recreation activities because of pain in my neck
- I can’t do any recreation activities at all

**PHYSICAL ACTIVITY**

Please indicate, on average, how often you currently exercise:

|  | Every Day | 2-5 x / week | 1 x / week | 1-3 x / month | Less than 1 x / month | never |
| --- | --- | --- | --- | --- | --- | --- |
| Running or jogging |  |  |  |  |  |  |
| Cycling (including ergometer) |  |  |  |  |  |  |
| Swimming |  |  |  |  |  |  |
| Rowing (including ergometer) |  |  |  |  |  |  |
| Resistive exercises |  |  |  |  |  |  |
| Core stability training |  |  |  |  |  |  |
| Specific neck exercises |  |  |  |  |  |  |
| Ball games (basketball, tennis, soccer, hockey, etc…) |  |  |  |  |  |  |
| Other (specify; auto generate additional rows as needed) |  |  |  |  |  |  |

FREE TEXT ENTRY BOX FOR THE EXAMINER: Examiner to catalogue any further relevant information. I.e. more detailed subjective examination where pain/parathesia is present.
