# Supplementary material for: Cervical spine and muscle adaptation after spaceflight and relationship to herniation risk: protocol from ‘Cervical in Space’ trial
Source: BMC Musculoskelet Disord. 2022 Aug 13;23:772. doi: 10.1186/s12891-022-05684-0 (PMC9375326; doi:10.1186/s12891-022-05684-0)
Supplement: Supplementary file 3 — Additional file 3. [file 12891_2022_5684_MOESM3_ESM.docx]

Supplementary Material 3: Contributor Roles Taxonomy (CRediT) <https://casrai.org/credit/>

| **Term** | **Who?** | **Definition** |
| --- | --- | --- |
| Conceptualization | DLB, GA, KA, HB, DF, RS, RS, HJW, KR | Ideas; formulation or evolution of overarching research goals and aims |
| Methodology | DLB, GA, KA, HB, DF, RS, RS, HJW, KR | Development or design of methodology; creation of models |
| Software | NA (protocol manuscript) | Programming, software development; designing computer programs; implementation of the computer code and supporting algorithms; testing of existing code components |
| Validation | NA | Verification, whether as a part of the activity or separate, of the overall replication/ reproducibility of results/experiments and other research outputs |
| Formal analysis | NA (protocol manuscript) | Application of statistical, mathematical, computational, or other formal techniques to analyze or synthesize study data |
| Investigation | NA (protocol manuscript) | Conducting a research and investigation process, specifically performing the experiments, or data/evidence collection |
| Resources | DLB, GA, KA, HB, DF, RS, RS, HJW, KR | Provision of study materials, reagents, materials, patients, laboratory samples, animals, instrumentation, computing resources, or other analysis tools |
| Data Curation | NA (protocol manuscript) | Management activities to annotate (produce metadata), scrub data and maintain research data (including software code, where it is necessary for interpreting the data itself) for initial use and later reuse |
| Writing - Original Draft | DLB, NKA, VK, SK | Preparation, creation and/or presentation of the published work, specifically writing the initial draft (including substantive translation) |
| Writing - Review & Editing | DLB, NKA, VK, SK, GA, KA, HB, DF, RS, RS, HJW, KR, EMV, MA, FG, BB | Preparation, creation and/or presentation of the published work by those from the original research group, specifically critical review, commentary or revision – including pre-or postpublication stages |
| Visualization | NA (protocol manuscript) | Preparation, creation and/or presentation of the published work, specifically visualization/ data presentation |
| Supervision | DLB, GA, KA, DF, HB, KR | Oversight and leadership responsibility for the research activity planning and execution, including mentorship external to the core team |
| Project administration | DLB, VK, SK, NKA | Management and coordination responsibility for the research activity planning and execution |
| Funding acquisition | DLB, GA, KA, HB, DF, RS, RS, HJW, KR | Acquisition of the financial support for the project leading to this publication |
